# Retraction: Association of CYP2A6*4 with Susceptibility of Lung Cancer: A Meta-Analysis

**DOI:** 10.1371/journal.pone.0160299

**Published:** 2016-07-26

**Authors:** The PLOS ONE Editors

Lishan Wang requested the retraction of this article.

Upon review of the submission history for the manuscript, the *PLOS ONE* Editors found indications that the peer review process was compromised by the submission of reviews through fabricated reviewer accounts. An institutional investigation, conducted by the Administration Office of Bio-X Institutes at Shanghai Jiao Tong University, supported the concerns about the integrity of the review process and indicated that Lishan Wang was solely responsible for the article, contrary to information provided to the journal at time of submission.

In light of the above concerns, the *PLOS ONE* Editors retracted the research article [1] on July 26, 2016.

This retraction notice and the linked article [1] and Correction [2] were republished on June 7, 2022 to amend content, references, and metadata.

## References

[1] Wang L. (2013) Association of *CYP2A6*4* with Susceptibility of Lung Cancer: A Meta-Analysis. PLoS ONE 8(4): e59556. doi: 10.1371/journal.pone.0059556 23585826 PMC3622000

[2] Wang L. (2013) Correction: Association of *CYP2A6*4* with Susceptibility of Lung Cancer: A Meta-Analysis. PLoS ONE 8(8): doi: 10.1371/annotation/1e88e5e7-a6f9-426b-b9c5-26fc923a23c2 PMC3622000 23585826

